# Porcine reproductive and respiratory virus 2 infection of the fetus results in multi-organ cell cycle suppression

**DOI:** 10.1186/s13567-022-01030-3

**Published:** 2022-02-21

**Authors:** Margaret K. Mulligan, Jocelyn E. Kleiman, Andrew C. Caldemeyer, John C. S. Harding, J. Alex Pasternak

**Affiliations:** 1 Department of Animal Sciences, Purdue University, 915 W. State St., West Lafayette, IN 47907 USA; 2 Department of Large Animal Clinical Sciences, Western College of Veterinary Medicine, University of Saskatchewan, 52 Campus Dr., Saskatoon, SK S7N 5B4 Canada

**Keywords:** PRRS, host–pathogen interaction, maternal, fetal, hypothyroidism, cell cycle progression

## Abstract

Porcine reproductive and respiratory syndrome virus (PRRSV) infection during late gestation negatively affects fetal development. The objective of this study was to identify the fetal organs most severely impacted following infection, and evaluate the relationship between this response and fetal phenotypes. RNA was extracted from fetal heart, liver, lung, thymus, kidney, spleen, and loin muscle, collected following late gestation viral challenge of pregnant gilts. Initially, gene expression for three cell cycle promoters (CDK1, CDK2, CDK4) and one inhibitor (CDKN1A) were evaluated in biologically extreme phenotypic subsets including gestational age-matched controls (CON), uninfected (UNIF), high-viral load viable (HV-VIA), and high-viral load meconium-stained (HV-MEC) fetuses. There were no differences between CON and UNIF groups for any gene, indicating no impact of maternal infection alone. Relative to CON, high-viral load (HV-VIA, HV-MEC) fetuses showed significant downregulation of at least one CDK gene in all tissues except liver, while CDKN1A was upregulated in all tissues except muscle, with the heart and kidney most severely impacted. Subsequent evaluation of additional genes known to be upregulated following activation of P53 or TGFb/SMAD signaling cascades indicated neither pathway was responsible for the observed increase in CDKN1A. Finally, analysis of heart and kidney from a larger unselected population of infected fetuses from the same animal study showed that serum thyroxin and viral load were highly correlated with the expression of CDKN1A in both tissues. Collectively these results demonstrate the widespread suppression in cell division across all tissues in PRRSV infected fetuses and indicate a non-canonical regulatory mechanism.

**Supplementary Information:**

The online version contains supplementary material available at 10.1186/s13567-022-01030-3.

## Introduction

Porcine reproductive and respiratory syndrome virus (PRRSV) initially emerged in the late 1980s and has since become one of the most economically devastating pathogens affecting swine production. Classified into the Nidovirales order, PRRSV has a genome comprised of single stranded RNA, but unlike coronaviruses, lacks 3′exonuclease activity [1]. Consequently, the virus exhibits an exceptionally high mutation rate, with roughly one mutation expected per viral replication cycle [2], resulting in an ongoing re-emergence of novel strains. In combination with a well-established capacity for immune evasion [3], the PRRS virus is particularly difficult to control via classical methods such as vaccination or biosecurity. As its name suggests, PRRSV is of particular concern within the breeding herd, where it can lead to abortion and a significant increase in the number of stillborn and weak piglets in pregnancies carried to term. These negative reproductive outcomes arise following vertical transmission of the virus across the normally restrictive epitheliochorial placenta. However, the fetal impact following maternal infection is heterogeneous, and a meaningful portion (17.5–21.7% based on past challenge trials) of fetuses remain entirely uninfected, indicative of their resistance relative to their cohorts [4–6]. Once across the placental barrier of less resistant fetuses, the PRRS virus infects circulating monocytes and tissue resident macrophages present in a wide array of fetal organs [7], with the fetal thymus generally recognized as a primary site of viral replication [8]. Among the population of infected fetuses, a portion demonstrate significant resilience and remain viable despite extremely high viral loads in serum and thymic tissue [5, 6]. Conversely, the susceptible fetuses lose viability, and while the most susceptible fetuses are found dead and autolyzed shortly after maternal infection [9], a portion will be found alive but meconium stained, indicating the initial stages of fetal compromise.

The porcine fetus becomes susceptible to transplacental PRRSV infection starting as early as 70 days into a typical 114 day gestation period [10]. Unfortunately, this stage of gestation also corresponds with the period during which the fetus experiences the highest rate of fetal growth [11], resulting in significant energetic strain on the fetus. Fetal weight, as well as the weight of individual organs including the liver, heart, and kidney, increases at an exponential rate throughout the 114 day gestation period [12]. This growth is driven by progressive increases in growth promoting signals such as insulin-like growth factor 1 [13], and thyroid hormones [14] including thyroxin (T4) and its more bioactive derivative triiodothyronine (T3). Although the PRRSV infected fetus is capable of mounting an immune response [6, 8], it creates not only supplemental energetic cost but results in a cytokine response that may interfere with normal developmental processes, including a significant disruption in fetal thyroid hormone levels [15]. Moreover, type I and II interferons, which are elevated following fetal PRRSV expression [6], have a detrimental impact on placental and fetal development and are implicated in intrauterine growth restriction (IUGR) in other models of congenital infection [16, 17].

The late gestation surge in fetal growth is also coincident with a transition from cellular hyperplasia to hypertrophy which is part of the organ-specific development program required to prepare the fetus for extra-uterine survival. The process of cell division in highly conserved and tightly regulated in mammalian cells and can be divided into four sequential stages referred to as G1 (Gap1), S (DNA synthesis), G2 (gap 2), and M (mitosis) phases. Progression between these phases is largely controlled by a group of cyclic dependent kinases including CDK1, CDK2, and CDK4. Entry into the G1 phase is promoted by the activity of CDK4, which then functions in tandem with CDK2 to regulate entry into and out of the S phase. In turn, CDK1 drives progression through the G2 phase and into the M phase to complete the cell cycle. The activity of all three of these cell cycle promoters is suppressed by the cyclic dependent kinase inhibitor 1A (CDKN1A) more commonly known as P21 [18]. Inhibition of the cell cycle by CDKN1A is a component of the cellular stress response and is intimately entwined with apoptosis through the P53 signaling cascade [19]. This system is also known to be regulated in a P53 independent manner by the transforming growth factor beta (TGFβ) superfamily, via a signaling cascade involving the corresponding transmembrane receptor and SMAD proteins [20]. We have previously observed alteration in the expression of these genes in a subset of fetal organs including the thymus at 12 days post maternal infection (dpi) and the heart and brain at 21 dpi [6, 15]. As each fetal organ system follows a unique developmental trajectory, a more complete understanding of disruption in this system is required. To this end, the present study evaluated expression of these key regulatory genes across seven fetal organs, including heart (HRT), kidney (KID), spleen (SPLN), thymus (THY), liver (LVR), lung (LNG) and longissimus dorsi as a representative skeletal muscle (MUS). We then investigated the potential regulation of this system through canonical signaling pathways by evaluating expression of additional P53 and TGFβ/SMAD regulated genes. Finally, we evaluated the correlation between expression of CDKN1A and fetal phenotypes including viral load, thyroid hormones, and morphometric measurements.

## Materials and methods

### Challenge model

Fetal tissue samples were derived from a PRRSV challenge model involving late gestation pregnant gilts, which has been previously reported [4]. In short, 27 Yorkshire gilts, from a PRRSV-free nucleus herd (Fast Genetics, Saskatoon, Canada), were synchronized in 6 batches with oral progestogen (15 mg Altrenogest, Merck Animal Health, Kenilworth, USA) treatment for 14 days, and 36 h after withdrawal were treated with 800 IU pregnant mare serum gonadotropin (PMSG; Folligon, Merck Animal Health) before single sire artificial insemination with one of six Landrace sires. At 86 days of gestation 22 gilts, in 5 batches, were challenged with a total dose of 1 × 10^5^ TCID_50_ NVSL 97–7895, with half delivered via intramuscular injection and half delivered by intranasal atomization. A single batch of 5 gilts were similarly sham inoculated with minimum essential media, to serve as gestation aged matched controls. At 21 dpi, all animals were euthanized by intravenous barbiturate overdose. The gravid uterus was removed and linearized to allow for careful dissection and conservation of fetal and placental pairings. Developmentally normal fetuses, exhibiting blood pulsations within the umbilical cord were considered as live at the point of euthanasia and those with normal skin colouring were further categorized as viable (VIA). A second group of live fetuses were classified based on the presence and degree of meconium staining in the form of inspissated yellow–brown material on either the face alone or the more severe face and body (MEC). Fetal blood was immediately collected from the axillary artery and serum later separated and stored at −80 °C. Select fetal organs including brain, LVR and HRT were weighed, and the left and right fetal profile imaged for later analysis of crown rump length (CRL) using a semi automated image analysis macro. Fetal tissue samples including the tip of the right middle lobe of LNG, a marginal piece of LVR, a combination of cervical and thoracic THY, the apex of the HRT, and central cross sections of the KID, SPLN and MUS. The pregnant gilt challenge model was carried out in strict accordance with the guidelines of the Canadian Council of Animal Care and with approval of the University of Saskatchewan’s Animal Research Ethics Board (Protocol #20,180,071).

### Fetal selection

Volume or tissue weight normalized PRRSV RNA concentration (target copies/µL or mg) was assessed in fetal serum and thymus as extensively described elsewhere [5, 21]. In short, total RNA was extracted and viral RNA quantified using primer and probe sequences targeting the distal region of ORF7 and a one-step qPCR assay including a standard curve comprised of linearized plasmid. Fetal serum thyroid hormone levels (T3 and T4) were each evaluated using commercially available RIA as previously described [15]. For initial evaluation, the resulting data on viral load and thyroid hormone level was used in combination with meconium staining to identify subsets of fetuses from the larger population produced by the challenge model that represent four biologically distinct, and physiologically extreme groups. Viable fetuses from PRRSV inoculated dams with no detectable viral RNA in either serum or thymus were classified as uninfected (UNIF). Fetuses with > 7 log_10_/µL and 5 log_10_/mg in serum and thymus, respectively, were categorized as high viral load and further subdivided into resilient fetuses [13] that remained viable despite high viral load (HV-VIA), and susceptible fetuses who exhibited both high viral load and severe meconium staining of the face and body (HV-MEC), a marker of fetal compromise. Finally, control (CON) fetuses were selected from the litters of gestation day matched, mock-inoculated gilts. Within each phenotypic fetal group, z-scores for fetal T4 were calculated and *n = *10 fetuses with the lowest z-scores in each group selected for in-depth study. To identify potential relationships between previously established phenotypic factors including viral load in serum, placenta and THY, circulating T3 and T4, and fetal morphometrics, cDNA collections for HRT and KID were subsequently expanded to include all available fetuses with confirmed viral load from which high quality RNA could be isolated, resulting in cDNA from a total of 120 HRT and 114 KID.

### Genes expression analysis

**Figure 1:**
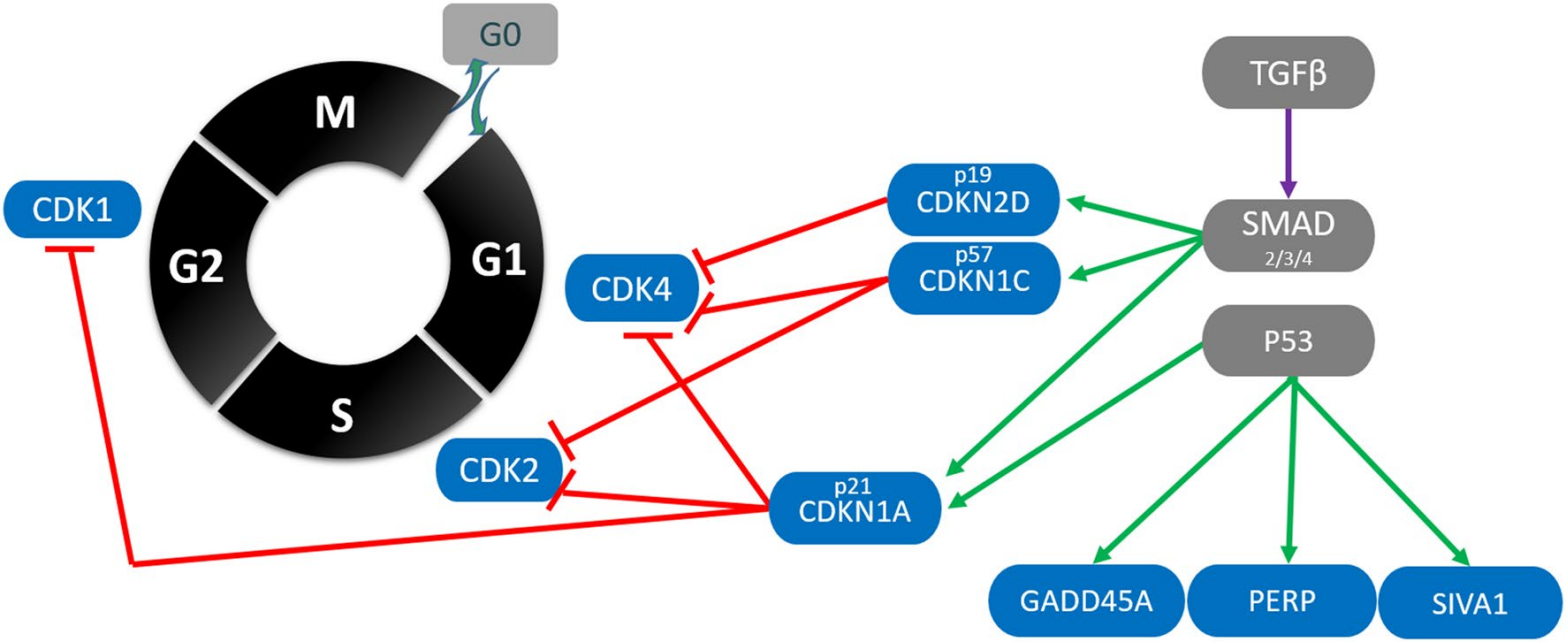
**Schematic representation of interrelationship between genes of interest.** Target genes (purple) with canonical regulatory pathways (grey). Blue arrows indicate established transcriptional activation, red and green indicate activation and inhibitory protein–protein interactions, respectively.

Samples of HRT, LVR, KID, LNG, SPLN, MUS, and THY, from selected fetuses, were ground to a fine powder under liquid nitrogen in a mortar and pestle. Total RNA was then isolated using Trizol (Thermofisher Scientific, Waltham, USA) and a double precipitation method as previously described [22] with DNA contamination removed using the turbo DNase protocol (Thermofisher Scientific). RNA quantity was determined using a Nanodrop spectrophotometer (Thermofisher Scientific) and integrity was assessed using denaturing agarose gel electrophoresis [23]. Finally, the High Capacity cDNA Reverse Transcription kit (Thermofisher Scientific) was used to generate cDNA from 2 μg of DNA free total RNA. Gene-specific primers were designed or modified to capture all currently predicted transcript variants for target RefSeq mRNA sequences (Table 1) corresponding to 5 housekeeping genes and 9 genes of interest either directly regulating cell cycle progression or transcriptionally regulated by P53 or TGFb/SMAD (Figure 1). Where possible primers were positioned to span exon–exon junctions, identified by the BLAST-like alignment tool (BLAT), against the *Sus scrofa* 11.1 genome assembly. Primer efficiency for each target was determined to be greater than 90% and melting curve analysis suggested a single amplicon product. Real time PCR was carried out in duplicate on 20 ng cDNA using the Sso advanced universal sybr green supermix (BioRad, Hercules, USA) and CFX qPCR system (BioRad). The geometric mean of the two most stable housekeeping genes for each tissue was then used to normalize expression data within the respective tissue. The resulting expression data are presented in the form of fold changes relative to the average expression of the CON group within tissues using the 2^−ΔΔCT^ method with fold changes reported based on the average of the group in question.

**Table 1:** **Porcine specific primer sequences used for qPCR**

	Gene ID	Symbol	Forward primer	Reverse primer	Amplicon length	Annealing Temp
Housekeeping	414,396	ACTB	5′-CCAGCACGATGAAGATCAAG-3′	5′-AGTCCGCCTAGAAGCATTTG-3′	171	60
	396,581	HMBS	5′-AGGATGGGCAACTCTACCTG -3′	5′-GATGGTGGCCTGCATAGTCT-3′	83	61
	397,637	PPIA	5′-CACTGCCAAGACTGAGTGGT-3′	5′-TGTCCACAGTCAGCAATGGT-3′	144	61
	780,433	SDHA	5′-CTACAAGGGGCAGGTTCTGA-3′	5′-AAGACAACGAGGTCCAGGAG-3′	141	61
GOI	100,628,048	STX5	5′-TGCAGAGTCGTCAGAATGGA-3′	5′-CCAGGATTGTCAGCTTCTCC-3′	144	60
	100,155,762	CDK1	5′-CAGCTCGCTACTCAACTCCA-3′	5′-GAGTGCCCAAAGCTCTGAAA-3′	135	61
	100,154,715	CDK2	5′-CGGAGCTTGTTATCGCAAAT-3′	5′-AGGGGTAGGGTTCACAAAGG-3′	143	61
	100,144,492	CDK4	5′-TGGTTACAAGTGGTGGGACA-3′	5′-CCACAGAAGAGAGGCTTTCG-3′	208	61
	100,152,215	CDKN1A	5′-CATGTGGACCTGTTGCTGTC-3′	5′-TTAGGGCTTCCTCTTGGAGA-3′	168	61
	110,259,225	CDKN1C	5′-ACGACACAGCGAACGAGAC-3′	5′-GCAGCTCACGACTCAGCTC-3′	164	60
	100,525,560	CDKN2D	5′-TGCTGCTAGAGGAGGTCTGC-3′	5′-CTGCCAAACATCATGACCTG-3′	157	61
	733,669	GADD45A	5′-ACGCCGCTCTCTCTCAGTAG-3′	5′-CCCCACCTTATCCATCCTTT-3′	98	59
	100,513,507	PERP	5′-TGGTGGAAGTGTTCTCAGGA-3′	5′-ACTCGCAGGAAGACAAGCAT-3′	188	61
	110,258,343	SIVA1	5′-CGCTACAGCTCAAGGTTCG-3′	5′-GGGTGGTCTTCTCGAAAATCT-3′	93	60

### Statistical analyses

All data processing and analyses were carried out in R 3.6.1 [24]. Gene expression data was found to be largely non-normal and was therefore assessed for all genes using a non-parametric methodology consisting of Kruskal–Wallis test followed by post-hoc pairwise comparisons using the Wilcoxon rank sum test with the resulting *P* value adjusted for multiple comparisons using the Bonferroni correction. Data was visualized using the ggplot2 package [25] with observed statistical differences (*P* < 0.05), where present, marked with unique superscripts. Pearson’s product-moment correlation was used to assess the linear correlation between continuous phenotypic variables and CDKN1A fold change relative to control fetuses in extended cDNA collections for HRT and KID. The strength of each correlation was then categorized using an established system [26].

## Results

### Expression of cell cycle promoters across seven fetal organs

**Figure 2:**
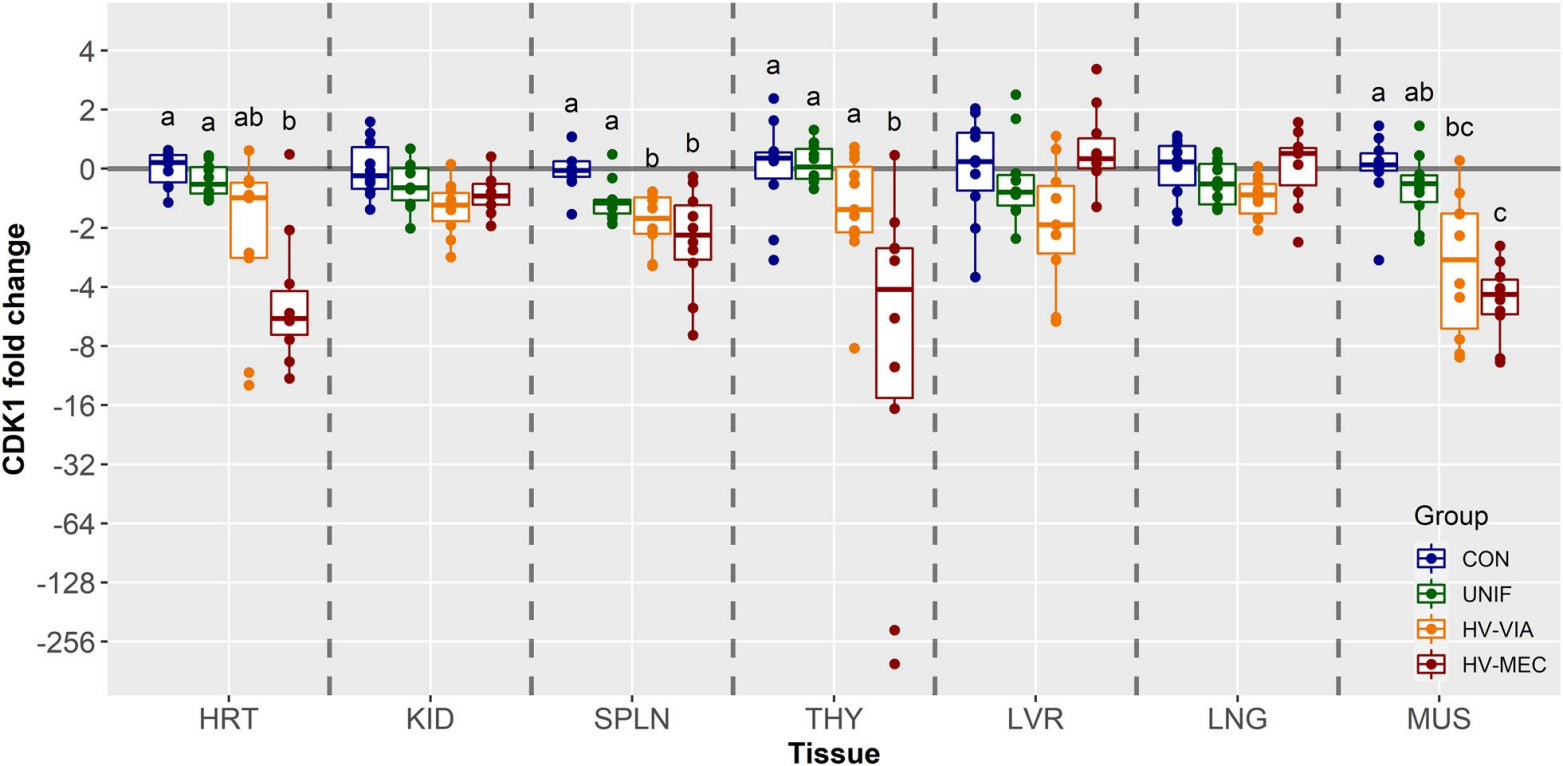
**Expression of cell cycle promoter CDK1.** CDK4 gene expression in fetal tissues including the heart (HRT), kidney (KID), spleen (SPLN), liver (LVR), lung (LNG), thymus (THY), and loin muscle (MUS) derived from fetuses collected from PRRSV-2 challenged dams at 21 days post-inoculation and classified based on viral load in serum and thymus as uninfected (UNIF, *n* = 10), high viral load viable (HV-VIA, *n* = 10) or high viral load meconium stained (HV-MEC, *n* = 10), or from control fetuses (CON *n* = 10) collected from gestation day matched non-inoculated control gilts. Fold changes were calculated within tissue relative to the average of the CON group with different superscripts denoting statistical differences (*P* < 0.05) within a given tissue.

To understand the impact of fetal PRRSV infection on late gestation organ development, we evaluated expression of three cell cycle promoters (CDK1, 2, and 4) and the suppressor CDKN1A across seven fetal tissues derived from established fetal phenotypes. On average, expression of CDK1, which promotes transition from S to G2 and G2 to M phases, was greatest in SPLN followed sequentially by THY, HRT, LVR, KID, LNG, MUS (Additional file 1). CDK1 showed no significant group differences in KID, LVR, or LNG or between CON and UNIF fetuses for the remaining four organs (Figure 2). Expression of CDK1 was significantly downregulated in HV-MEC fetuses in both HRT (x̃ =  −5.79 fold, *P* = 0.008) and THY (x̃ =  −3.89 fold, *P* = 0.022) relative to control, but in both tissues the comparative numerical decrease in HV-VIA fetuses versus CON was found to be non-significant (*P* = 0.149 and *P* = 0.269 respectively). In SPLN, a significant decrease in expression of CDK1 was observed in both HV-VIA (x̃ =  −1.58 fold, *P* = 0.076) and HV-MEC (x̃ =  −2.16 fold, *P* = 0.076) fetuses relative to CON. The expression pattern was similar in MUS, with HV-VIA (x̃ =  −2.79 fold, *P* = 0.022) and HV-MEC (x̃ =  −4.36 fold, *P* = 0.002) both significantly depressed relative to CON.

**Figure 3:**
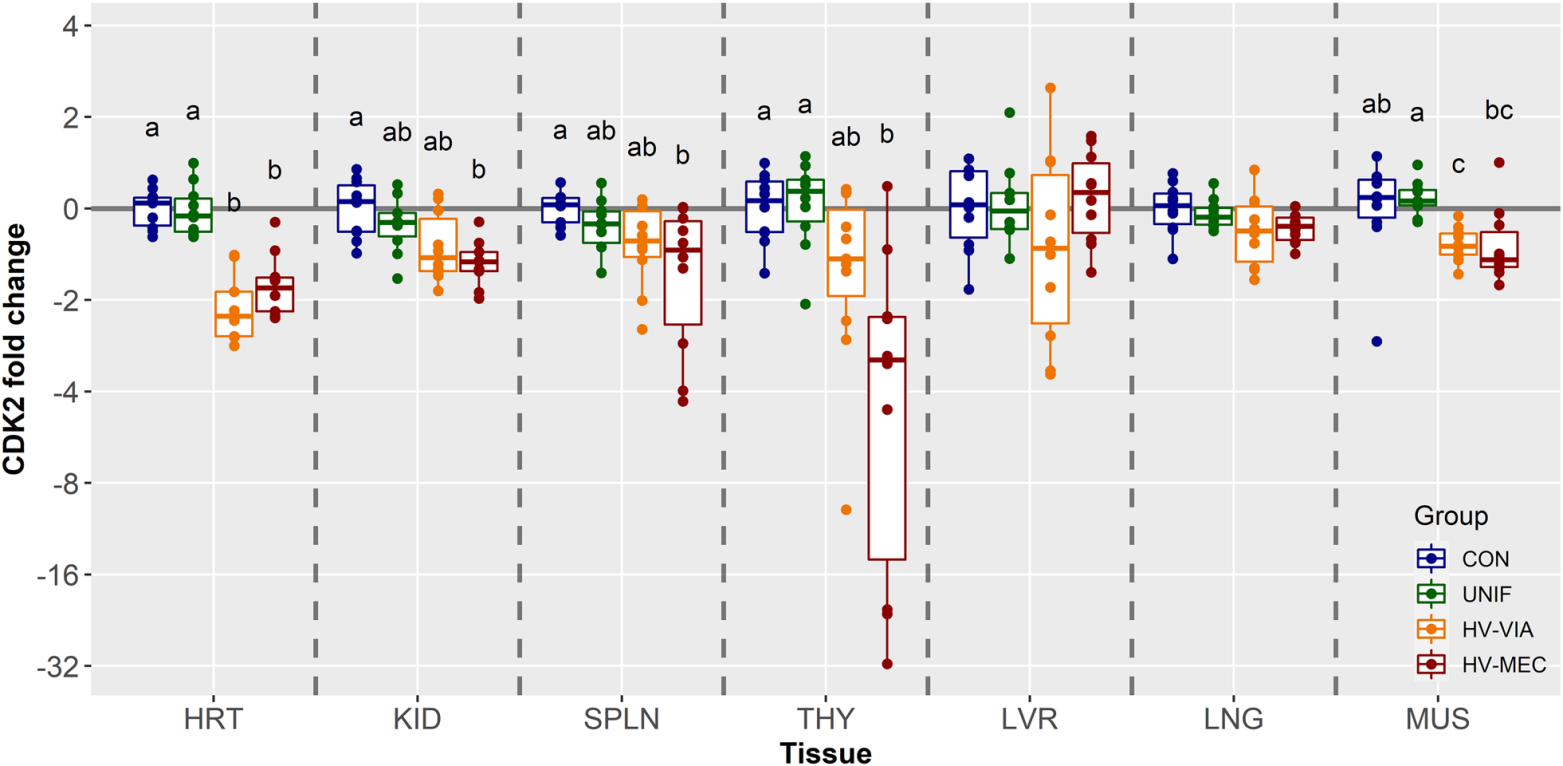
**Expression of cell cycle promoter CDK2.** CDK2 gene expression in fetal tissues including the heart (HRT), kidney (KID), spleen (SPLN), liver (LVR), lung (LNG), thymus (THY), and loin muscle (MUS) derived from fetuses collected from PRRSV-2 challenged dams at 21 days post-inoculation and classified based on viral load in serum and thymus as uninfected (UNIF, *n* = 10), high viral load viable (HV-VIA, *n* = 10) or high viral load meconium stained (HV-MEC, *n* = 10), or from control fetuses (CON *n* = 10) collected from gestation day matched non-inoculated control gilts. Fold changes were calculated within tissue relative to the average of the CON group with different superscripts denoting statistical differences (*P* < 0.05) within a given tissue.

Analysis of CDK2, which is required for cells to progress out of the G1 and into the S phase of the cell cycle, suggests the highest expression in SPLN and THY followed by HRT, LNG, KID, and LVR, with the lowest expression observed in MUS (Additional file 1). Similar to CDK1, analysis of CDK2 expression by fetal phenotype (Figure 3) revealed no significant difference in expression in LVR or LNG, or between CON and UNIF fetuses in any of the seven tissues examined. Significant decreases in expression relative to CON were observed for HV-MEC fetuses in the KID (x̃ =  −1.49 fold, *P* = 0.010), SPLN (x̃ = −1.36 fold, *P* = 0.029), and THY (x̃ =  −3.14 fold, *P* = 0.010). No significant difference was observed for the HV-VIA group in the THY, while the numerical decrease in both KID and SPLN trended toward significance (*P* = 0.096) relative to control. In MUS, expression of CDK2 was significantly decreased in HV-VIA fetuses relative to both CON (x̃ =  −1.33 fold, *P* = 0.028) and UNIF (x̃ =  −1.39 fold, *P* = 0.002), whereas HV-MEC fetuses only differed significantly (x̃ =  −1.53 fold, *P* = 0.028) from UNIF. Relative to both CON and UNIF, HRT tissues from highly infected fetuses showed significant suppression in CDK2, with median downregulations of −2.26 and −1.82 fold in HV-VIA (*P* = 0.017) and HV-MEC (*P* = 0.026) relative to CON.

**Figure 4:**
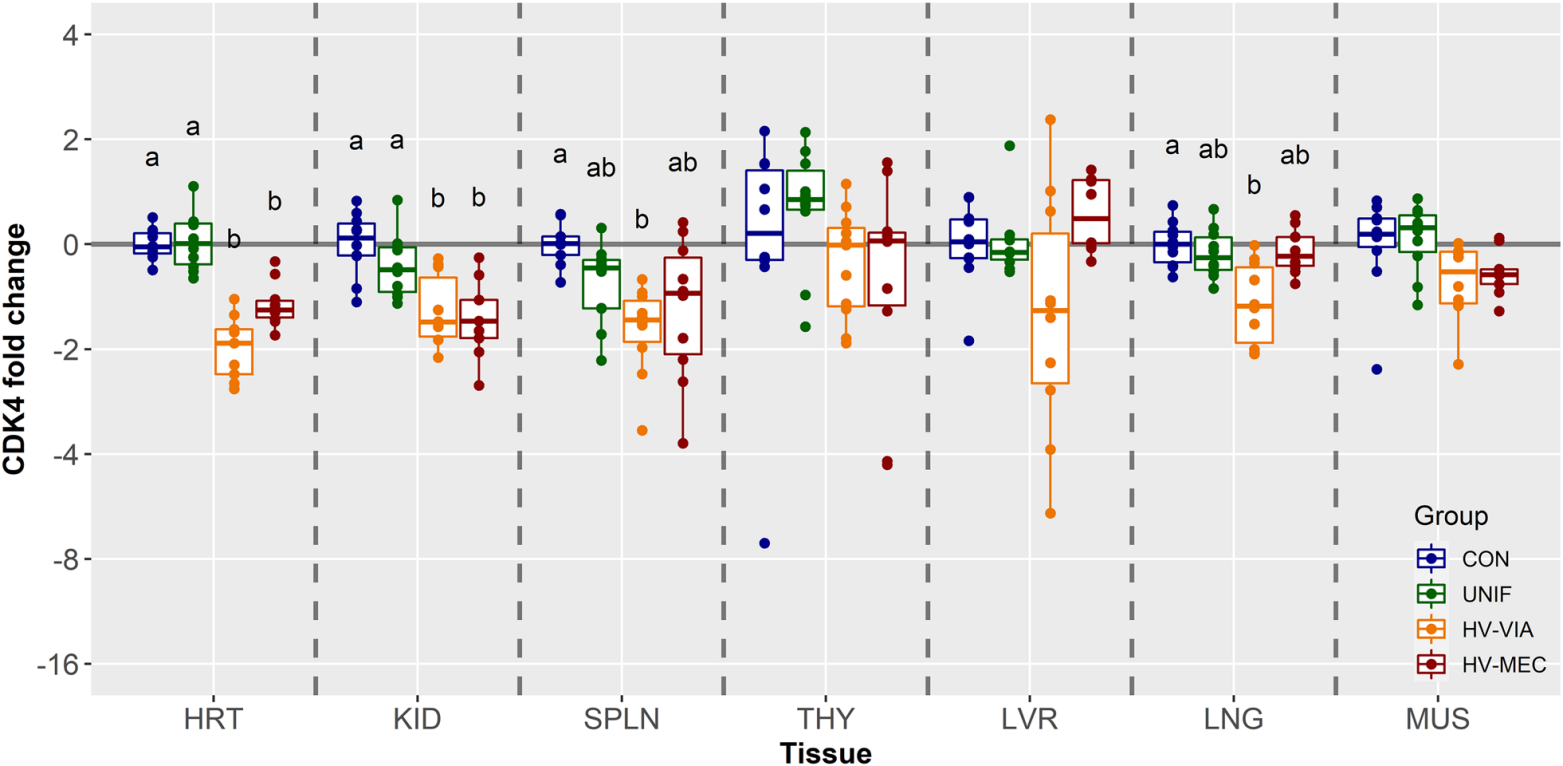
**Expression of cell cycle promoter CDK4.** CDK4 gene expression in fetal tissues including the heart (HRT), kidney (KID), spleen (SPLN), liver (LVR), lung (LNG), thymus (THY), and loin muscle (MUS) derived from fetuses collected from PRRSV-2 challenged dams at 21 days post-inoculation and classified based on viral load in serum and thymus as uninfected (UNIF, *n* = 10), high viral load viable (HV-VIA, *n* = 10) or high viral load meconium stained (HV-MEC, *n* = 10), or from control fetuses (CON *n* = 10) collected from gestation day matched non-inoculated control gilts. Fold changes were calculated within tissue relative to the average of the CON group with different superscripts denoting statistical differences (*P* < 0.05) within a given tissue.

Expression of CDK4, which also serves to promote the G1-S phase transition, was highest in SPLN and THY followed sequentially by KID, LNG, LVR, HRT, and MUS (Additional file 1). Again, analysis by phenotypic group showed no significant difference between CON and UNIF fetuses in any of the tissues evaluated (Figure 4). In addition, there were no significant differences observed between any of the four phenotypic groups in LVR, THY or MUS. Expression of CDK4 was significantly downregulated in HV-VIA relative to CON in the SPLN (x̃ =  −1.64 fold, *P* = 0.002) and LNG (x̃ =  −1.50 fold, *P* = 0.022), whereas no significant differences in these tissues were observed for HV-MEC fetuses. In the KID, there was significant suppression of CDK4 in both HV-VIA (x̃ =  −1.67 fold, *P* = 0.006) and HV-MEC (x̃ =  −1.66 fold, *P* = 0.008) fetuses relative to CON. Expression in the HRT showed a similar pattern with −1.92 and −1.54 fold decrease relative to CON observed in HV-VIA and HV-MEC fetuses, respectively (*P* = 0.002).

**Figure 5:**
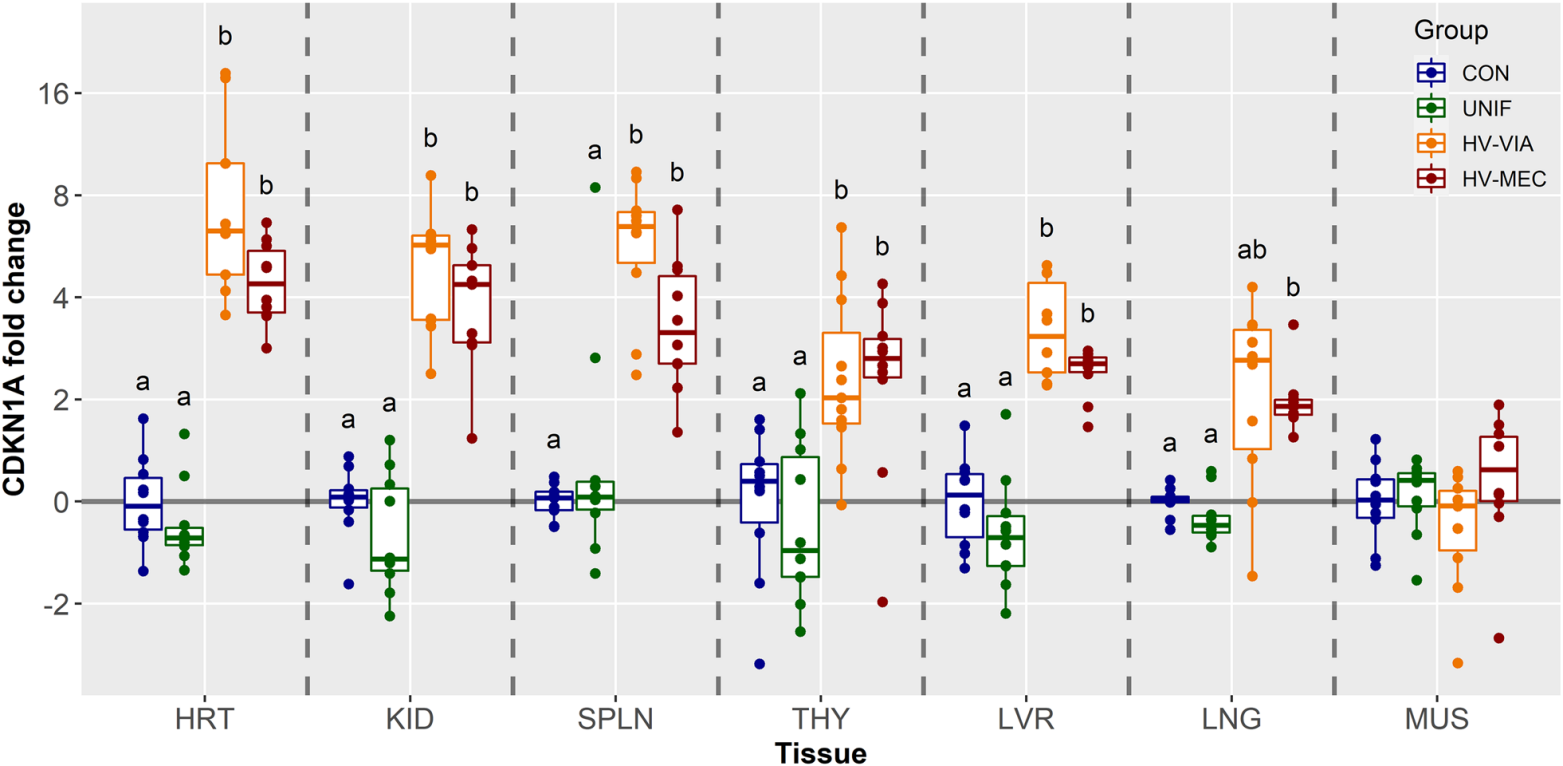
**Expression of cell cycle inhibitor CDKN1A.** CDKN1A gene expression in fetal tissues including the heart (HRT), kidney (KID), spleen (SPLN), liver (LVR), lung (LNG), thymus (THY), and loin muscle (MUS) derived from fetuses collected from PRRSV-2 challenged dams at 21 days post-inoculation and classified based on viral load in serum and thymus as uninfected (UNIF, *n* = 10), high viral load viable (HV-VIA, *n* = 10) or high viral load meconium stained (HV-MEC, *n* = 10), or from control fetuses (CON *n* = 10) collected from gestation day matched non-inoculated control gilts. Fold changes were calculated within tissue relative to the average of the CON group with different superscripts denoting statistical differences (*P* < 0.05) within a given tissue.

Finally, we evaluated expression of the potent cell cycle regulator CDKN1A, which was most abundant in MUS followed by LVR, LNG, HRT, KID/SPLN, and finally THY. Once again, we showed no significant difference in expression between CON and UNIF fetuses for any of the 7 tissues evaluated. In contrast, expression was significantly elevated among fetuses with high viral load in six out of seven tissues tested with the sole exception being muscle (Figure 5). The largest upregulation in CDKN1A was observed in HRT where median fold change relative to controls was 6.28 and 4.41 in HV-VIA (*P* = 0.001) and HV-MEC (*P* = 0.002) respectively. A similar pattern of expression with significant upregulation in both HV-VIA and HV-MEC was observed in four other tissues with median upregulation for all high viral load fetuses combined was greatest in SPLN (x̃ = 4.77 fold) followed closely by KID (x̃ = 4.72 fold), LVR (x̃ = 2.62 fold) and THY (x̃ = 2.4 fold). Expression of CDKN1A was significantly upregulated LNG from HV-MEC (x̃ =  −1.91 fold, *P* = 0.001), but not the HV-VIA (*P* = 0.104) fetuses.

### The role of canonical cell cycle regulation pathways

**Figure 6:**
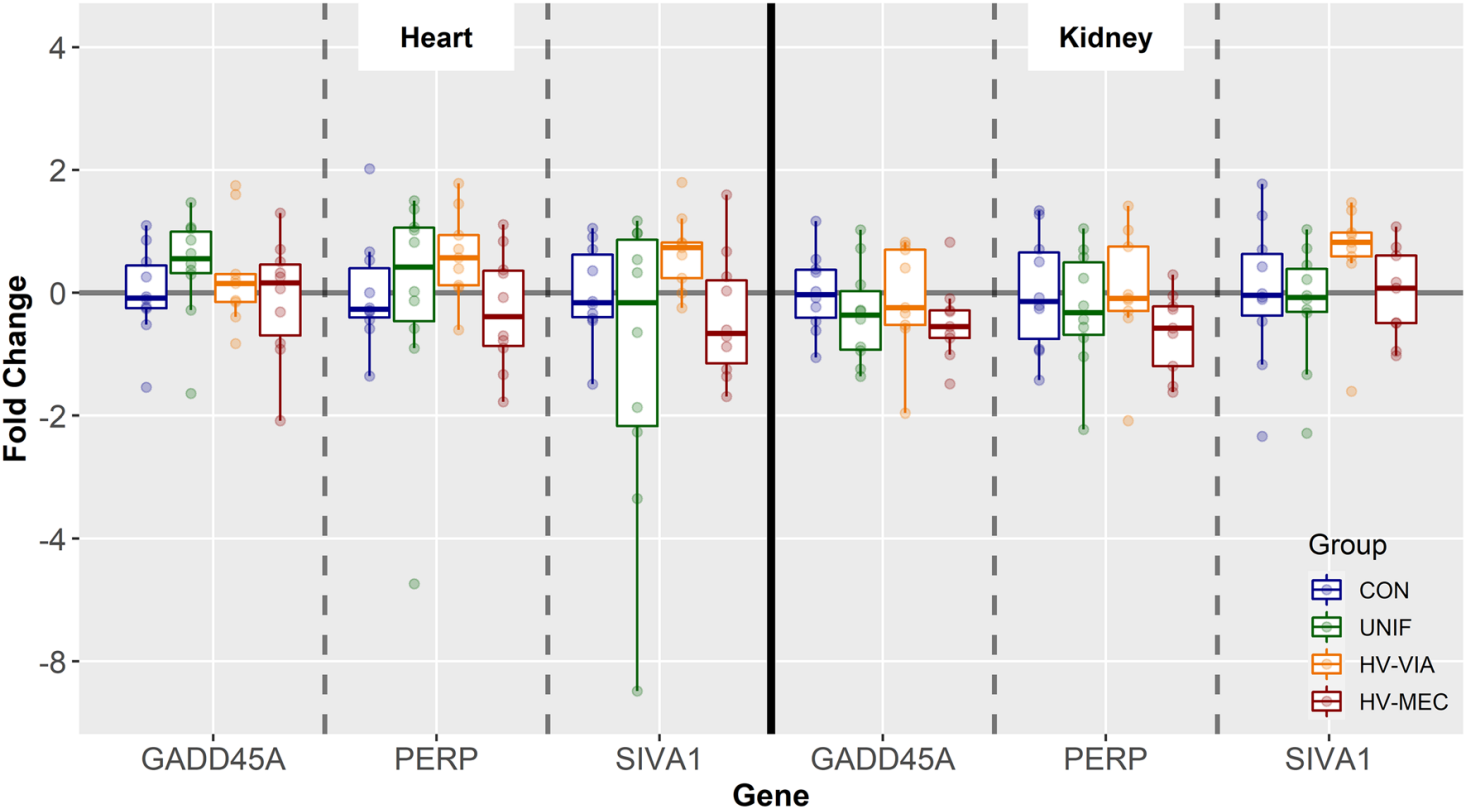
**Expression of genes known to be expressed following activation of the P53 signaling pathway.** Gene expression in the heart (HRT) and kidney (KID) derived from fetuses collected from PRRSV-2 challenged dams at 21 days post-inoculation and classified based on viral load in serum and thymus as uninfected (UNIF, *n* = 10), high viral load viable (HV-VIA, *n* = 10) or high viral load meconium stained (HV-MEC, *n* = 10), or from control fetuses (CON *n* = 10) collected from gestation day matched non-inoculated control gilts. Fold changes were calculated within tissue relative to the average of the CON group. No significant differences (*P* < 0.05) were detected in any of the three genes in either tissue.

**Figure 7:**
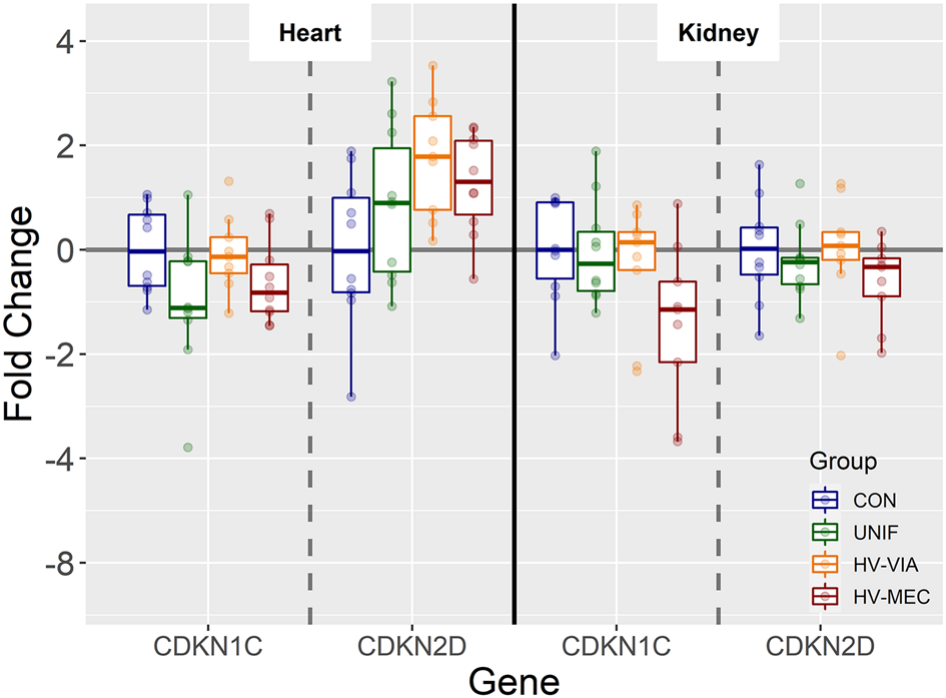
**Expression of genes known to be upregulated following activation of the TGFβ/SMAD signaling pathway.** Expression of CDKN1C and CDKN2Din the heart (HRT) and kidney (KID) derived from fetuses collected from PRRSV-2 challenged dams at 21 days post-inoculation and classified based on viral load in serum and thymus as uninfected (UNIF, *n* = 10), high viral load viable (HV-VIA, *n* = 10) or high viral load meconium stained (HV-MEC, *n* = 10), or from control fetuses (CON *n* = 10) collected from gestation day matched non-inoculated control gilts. Fold changes were calculated within tissue relative to the average of the CON group. No significant differences (*P* < 0.05) were detected in any of the three genes in either tissue.

To evaluate the potential role of canonical signaling pathways in suppressing cell division in the PRRSV infected fetus, we focused our attention on the HRT and KID. We first evaluated expression of genes known to be upregulated following activation of P53 including GADD45A, PERP, and SIVA1. No significant differences in any of these genes were observed in either tissue evaluated (Figure 6). We then evaluated expression of other cell cycle suppressing genes known to be upregulated following activation of the TGFβ/SMAD signaling pathway including CDKN1C and CDKN2D. Similarly, no significant changes were identified for any of these genes in either the HRT or KID (Figure 7). To confirm these findings in lymphoid tissues, expression of all five genes was also evaluated in the SPLN (Additional file 2), where again no significant differences were observed.

### Relationship between CDKN1A expression and continuous fetal phenotypes

**Figure 8:**
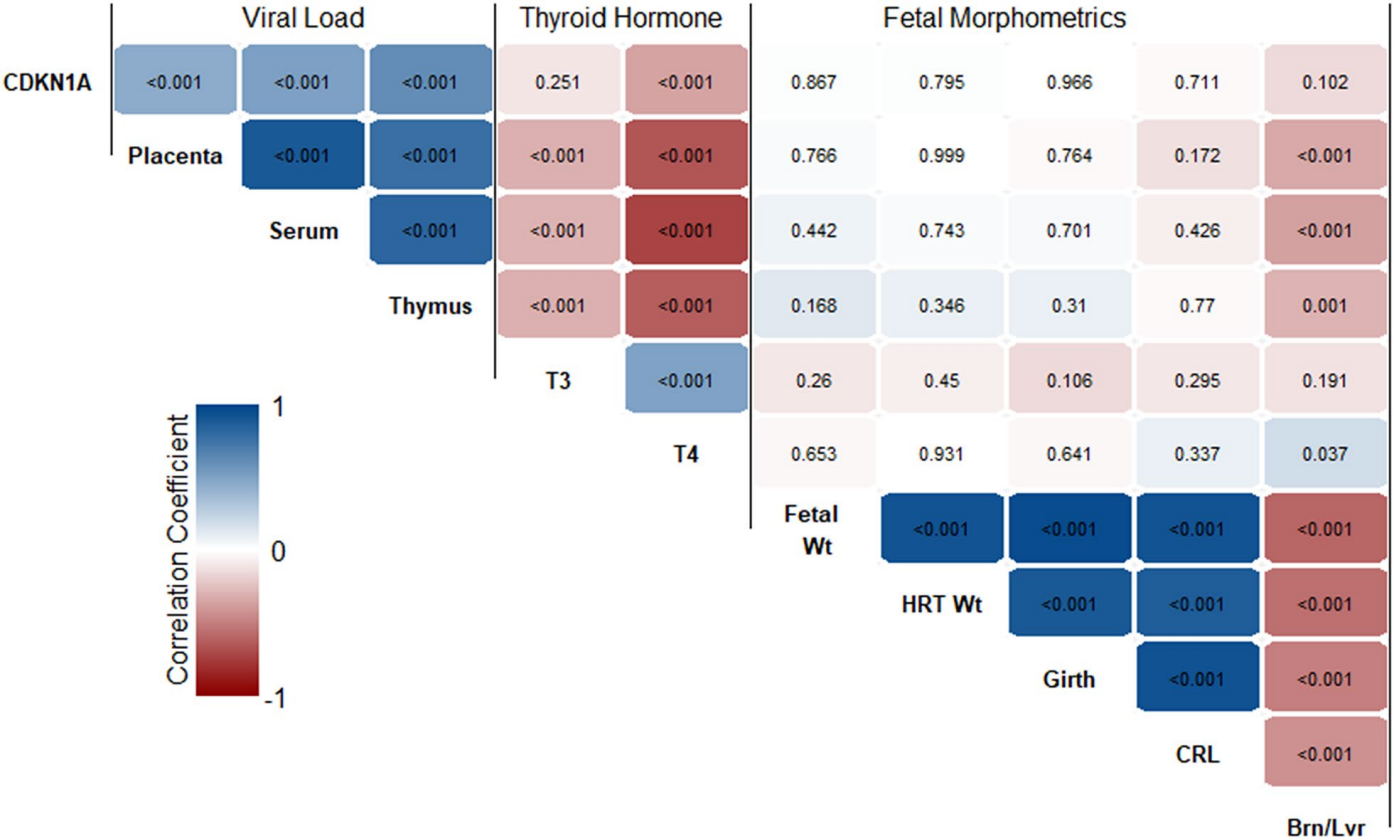
**Phenotypic correlations with cardiac CDKN1A expression.** Correlation matrix heat map demonstrating the interrelationships between expression of CDKN1A in heart tissue from *n* = 120 fetuses from PRRSV infected dams and phenotypic parameters including viral load (placenta, serum, and thymus), serum thyroid hormone concentration (T3 and T4) and fetal morphometrics. Pearson correlation coefficients for each comparison are indicated by colour with associated *P* value superimposed within each cell.

To identify potential relationships between previously established phenotypic factors including viral load, thyroid hormone and fetal morphometrics, existing cDNA collections for HRT and KID were expanded to include all available fetuses from the animal model with confirmed viral load greater than 0.5 log_10_ in both serum and thymus. We first evaluated the relationship between fold change in CDKN1A relative to control in HRT and KID and observed expression in the two tissues was moderate positive correlation (r = 0.58, *P* < 0.001). Next, we examined the relationship between cardiac expression of CDKN1A and various fetal phenotypes (Figure 8). Cardiac expression of CDKN1A was found to have low to moderate positive correlations with viral load in the placenta (r = 0.45, *P* < 0.001), serum (r = 0.51, *P* < 0.001) and thymus (r = 0.61, *P* < 0.001). In contrast, T4 showed a low negative correlation (r =  −0.36, *P* < 0.001) with CDKN1A expression, while no significant correlation was identified for T3. No significant correlation was identified between cardiac CDNK1A expression and fetal heart weight, or any other morphometric parameters. Unsurprisingly, the same pattern of correlations was found when evaluating renal expression of CDKN1A (Additional file 3).

## Discussion

This study has shown, for the first time, widespread disruption in the cell cycle regulation in fetal tissues following late gestation maternal PRRSV challenge. As with many of our previous studies [6, 15, 21, 27], the observed changes in gene expression were entirely restricted to highly infected fetuses. This apparent compartmentalization further demonstrates that the pathophysiological impact of PRRSV on the fetus is not a secondary effect of maternal infection, but rather the direct result of fetal infection. A finding that stands in contrast to farm level observations, where samples from aborted fetuses are often PRRSV negative [28], an incongruity which may be associated with either heterogeneous infection of the aborted litter or the difficulty associated with detecting viral RNA in sample collected under such conditions. Furthermore, the changes in gene expression in high viral load viable and compromised meconium stained fetuses were nearly identical, indicating the independence of cell cycle regulation from the factors associated with fetal death. Although all seven tissues evaluated showed significant alteration in gene expression associated with the regulation of cell division, the specific gene expression pattern differed between tissues. This variation is likely associated with the tissue-specific developmental processes underway during the challenge period. Furthermore, the impact of this disruption on fetal viability and post-natal survival and performance is tissue specific.

Of the tissues evaluated in the present study, gene expression in the HRT was the most severely impacted by infection, with significant decreases in all three cell cycle promoters (CDK1, CDK2 and CDK4) and the largest increase in expression of their inhibitor CDKN1A. This finding is consistent with our previous investigations which showed equivalent changes in cell division-associated gene expression in PRRSV infected fetuses just 12 days after maternal infection [15]. Such compromise of the heart is also consistent with the effects of other perturbations during fetal development such as intrauterine growth retardation, which places additional strain on the developing cardiovascular system, resulting in reduced maturation and increased apoptosis [29]. A decrease in the abundance of CDK2 and increase in CDKN1A has previously been associated with the transition of cardiomyocytes from hyperplasic to hypertrophic growth [30]. Interestingly, we observe no significant correlation between expression of CDKN1A and weight of the fetal HRT, which is consistent with previous analysis showing no significant change in HRT weight between various phenotypes of PRRSV infected fetuses [4]. In combination, these results may suggest that any PRRSV-induced decrease in cardiac hyperplasia may be countered by an increase in cellular hypertrophy associated with premature cardiac maturation.

The KID showed substantial disruption in cell cycle control, with a significant upregulation in CDKN1A and additional downregulation in CDK4 in highly infected groups. Unlike in humans, where nephrogenesis is completed by 36 weeks of gestation, the pig kidney continues to develop until roughly 3 weeks after parturition [31] suggesting active cell division in the normal pig KID during our late gestation viral challenge. The process of in utero nephrogenesis is known to be susceptible to multiple perturbations including maternal protein restriction [32], intrauterine growth retardation [33] and, congenital hypothyroidism [34]. However, the regulatory mechanism underlying renal maturation and the eventual cessation of nephrogenesis is not yet fully understood.

Terminal maturation of the fetal lung, including initiation of surfactant production and structural remolding to increase compliance, is a critical requirement for successful transition to extra uterine life and a by-product of the normal increase in fetal cortisol, which inevitably leads to parturition [35]. This glucocorticoid signal is also responsible for downregulating cell proliferation, which, if left unchecked, would lead to morphological abnormalities [36]. In the present study, we observed an upregulation in CDKN1A in HV-MEC and HV-VIA and a downregulation in CDK4 in the LNG of HV-VIA fetuses specifically. Similar to the HRT, this apparent suppression in cell division may be an indicator of premature pulmonary maturation, driven by an untimely stress response in the PRRSV infected fetus.

Hepatic tissue showed a significant increase in CDKN1A in highly infected fetuses, but all three CDK genes were found to be stable regardless of fetal infection status. The results in these tissues are similar to our previous findings in the brain, which we attribute to a well-known, but brain-specific, sparing effect [15]. Although hepatocytes undergo continuous proliferation throughout an organism’s life, fetal hepatocytes are programmed to proliferate at a higher rate than in the post-natal period [37]. Fetal hepatocytes are known to experience a temporary quiescence immediately prior to birth, which is coincident with the initiation of gluconeogenesis and the increase in glycogen required for parturition and initial post-natal survival. Interestingly, while post-natal hepatocytes exhibit the stereotypical inverse relationship between cell division and differentiation, proliferation in fetal hepatocytes appears to be disconnected from this terminal maturation process [38]. Rather, cortisol, which is responsible for initiating much of the terminal maturation in the fetus, has been shown to drive in vitro proliferation of fetal rat hepatocytes [37]. Thus, a fetal cortisol response alone cannot explain the observed increase in hepatic CDKN1A expression. Post-natal proliferation of hepatocytes has been shown to be dependent on thyroid hormone (T3) [39], suggesting the apparent downregulation in hepatocyte cell division may be the result of previously observed thyroid hormone suppression in the PRRSV infected fetus [15].

Piglets born alive following congenital infection with PRRSV experience high levels of post-natal mortality [40, 41], owing in part to a greater post-natal susceptibility to other pathogens [42]. While fetal hematopoiesis initially occurs within the liver before shifting to bone marrow later in gestation, some of the resulting cell populations, including T cells, are immature and require further development in secondary organs. T cell progenitors migrate to the thymus where they undergo both proliferation and maturation before being released into the peripheral population [43]. While not strictly dependent on a secondary tissue, B cells also undergo further differentiation and proliferation in the SPLN [44] where they subsequently produce a substantial quantity of circulating immunoglobulin. In addition, the SPLN has a direct role in immune responsiveness, functioning as both an antibody producing organ and a phagocytic filter for circulating bacteria. Of the seven tissues assessed in the present trial, the SPLN and THY showed an overall expression pattern most indicative of high proliferative activity. Consistent with our earlier investigation into this pathway in the PRRSV infected fetal THY [6], we observed altered regulation of cell proliferation in both SPLN and THY during a period of apparent rapid cell division. This result may partially explain the poor post-natal disease response and decrease in total circulating lymphocyte numbers in piglets congenitally infected with PRRSV [42].

The economic viability of swine production is dependent on the efficient post-natal growth of skeletal muscle. In the present study, MUS was unique among the tissues tested in that it showed no significant upregulation in CDKN1A in PRRSV-infected fetuses. This distinctive response is likely associated with the decreased hyperplasia during the challenge period, as fetal muscle fiber proliferation in the pig is thought to largely conclude between 85 and 95 days of gestation [45]. This late gestation shift from hyperplasia to hypertrophy, is consistent with the observed low expression of all three promoters (CDK1, 2 and 4) and highest expression of inhibitor (CDKN1A) relative to all other tissues assessed. As the fetal pig becomes susceptible to trans-placental PRRSV infection as early as 72 days of gestation [10, 46], the present challenge model with maternal infection at gestation day 86 may underestimate the impact of PRRSV on fetal muscle development. Despite the natural cessation in cell division, expression of CDK1 and CDK2 were still found to be further downregulated following fetal PRRS infection in the present study. Transcriptional regulation of cell cycle regulators in fetal muscle is known to continue in late gestation, with previous microarray studies showing decreased expression of CDK2 as well as CDK6 (now recognized as non-coding in the pig) and CDK9, between days 90 and day 110 [47]. Decreased expression of CDK1, CDK2, and CDK6 occurs during normal differentiation of myoblasts [48]. Thus, the observed decreased expression of CDK1 and CDK2 may indicate premature acceleration of fetal maturation in skeletal muscle following PRRSV infection. Muscle fiber number is fixed prior to birth, such that insufficient cell division during the fetal development will result in a permanent deficit in muscle mass which cannot be offset by compensatory post-natal growth [49]. As such any PRRSV induced suppression of in-utero skeletal muscle development would have significant impact of profitability and the impact of infection during earlier gestational stages warrants further investigation.

Expression of CDKN1A is canonically upregulated following activation of either the P53 [50] or TGFβ/SMAD [51] signaling pathways. In addition to CDKN1A, P53 mediated cell cycle arrest is known to include upregulation of GADD45A [52], which also serves to induce cell cycle arrest in the G1 stage. Similarly, both SIVA1 [53] and PERP [54] are upregulated following P53 activation as part of the apoptotic response pattern. The TGFβ/SMAD signaling cascade is known to result in the upregulation of additional cyclic dependent kinase inhibitors including CDKN1C (P57) [55] and CDKN2D (P19) [56]. In the present study, we observed no significant upregulation in any of co-regulated genes, suggesting that neither canonical pathway is responsible for the observed upregulation in CDKN1A. Under specific conditions, other signals such as IL1, retinoic acid, testosterone, or T3 have been shown to upregulated CDKN1A expression in a P53 independent manner [57, 58], suggesting that the observed changes in fetal gene expression may be the result of another signaling mechanism.

Thyroid hormones are a key regulator of fetal development, maturation and the surge in fetal mass during the later stages of gestation [59]. However, the role of this endocrine system in regulating cell division is unclear as it can exhibit both pro- [60, 61] and anti-proliferative effects [62]. The duality of this response in the developing fetus is exemplified in ovine thyroidectomy models which show increased proliferation in white adipose cells [63] and pancreatic beta cells [64], but decrease cardiomyocyte proliferation [65] in the absence of endogenous thyroid hormone. We have previously demonstrated that the PRRSV infected fetus experiences profound hypothyroidism following PRRSV infection [4, 15] which may be responsible for the observed changes in the regulation of cell division. Interestingly, the present study finds a significant negative correlation between both cardiac and renal CDKN1A expression and the serum concentration of T4, but not its more bioactive metabolite T3. The negative correlation with T4 would indicate further inhibition of cell division as the severity of PRRSV induced hypothyroidism increases. This relationship is however confounded during infection, by equally strong correlations between fetal viral load and both CDKN1A expression and thyroid hormone abundance. As a result, the cause and effect relationship between these factors cannot be established in the present experiments.

Cell division, which is a critical component of late gestation fetal development and the terminal maturation of vital organs, is disrupted following late gestation PRRSV challenge. Although varied in severity, we observe transcriptional evidence of suppressed cell cycle progression in seven major fetal organs. The degree of disruption in any given tissue may be linked to the timing of infection relative to the specific developmental program underway during the late gestation maturation of each organ system. The absence of observable changes in gene expression in any organ derived from uninfected fetuses is evidence that changes in fetal physiology are a result of fetal infection rather than a by-product of maternal infection. Interestingly, the observed changes in genes expression were not found to be associated with known canonical signaling pathways suggesting the presence of an as yet unidentified regulatory mechanism. While significant correlations with thyroid hormone are identified, further investigation is needed to determine the true cause and effect relationship and the post-natal implications of such a widespread disruption in the late gestation developmental program. Regardless of the regulatory mechanism, the results of this study clearly indicate that the PRRSV infected fetus is likely to suffer from post-natal dysfunction in multiple organ systems.

## Supplementary Information

**Additional file 1: Raw Expression data.** Average CT values across all phenotypic tissues across seven fetal organs including heart (HRT), kidney (KID), spleen (SPLN), liver (LVR), lung (LNG), thymus (THY) and loin muscle (MUS) derived from fetuses collected from Sham control and PRRS-2 challenged dams at gestation day 106 (21 days post maternal infection). Color indicates expression level within gene, with the highest expression coded green and lowest expression coded red.

**Additional file 2: Splenic Gene Expression.** Expression of genes known to be upregulated following activation of either P53 or TGFβ/SMAD signaling pathway in spleen derived from fetuses collected from PRRS-2 challenged dams at 21 days post-inoculation and classified based on viral load in serum and thymus as uninfected (UNIF, *n = *10), high viral load viable (HV-VIA, *n = *10) or high viral load meconium stained (HV-MEC, *n = *10), or from control fetuses (CON *n = *10) collected from gestation day matched non-inoculated control gilts. Fold changes were calculated within tissue relative to the average of the CON group. No significant differences (*P* < 0.05) were detected in any of the three genes in either tissue.

**Additional file 3: Phenotypic correlations with renal CDKN1A expression.** Correlation heat map demonstrating the interrelationship between expression of CDKN1A in kidney tissue from *n = *114 fetuses with some evidence of PRRSV infection and phenotypic parameter including viral load (placenta, serum and thymus), serum thyroid hormone concentration (T3 and T4) and fetal morphometrics. Pearson correlation coefficients were for each comparison are indicated by color with associated *P* value superimposed.

## Data Availability

The datasets generated during and/or analysed during the current study are available from the corresponding author on reasonable request.

## Abbreviations

CON: control fetuses collected from mock-infected gilts
dpi: days post maternal infection
G1: Gap1 phase of the cell cycle
G2: gap 2 phase of the cell cycle
HRT: fetal heart
HV-VIA: viable fetuses from PRRSV infected gilts with greater than 5 log at 21 dpi and 4.5 log at 12 dpi of virus in both fetal serum and thymus
HV-MEC: meconium stained fetuses from PRRSV infected gilts with greater than 5 log at 21 dpi and 4.5 log at 12 dpi of virus in both fetal serum and thymus
KID: fetal kidney
LNG: fetal lung
LVR: fetal liver
M: terminal mitosis phase of the cell cycle
MUS: fetal loin muscle
PRRSV: porcine reproductive and respiratory syndrome virus 2 (Strain NVSL 97–7895)
S: DNA synthesis stage of the cell cycle
SPLN: fetal spleen
THY: fetal thymus
T4: thyroxin
T3: triiodothyronine
TGFβ: transforming growth factor beta
UNIF: viable fetuses from PRRSV infected gilts with no virus detectable in fetal serum or thymus
