# Supplementary figures and images for: Porcine reproductive and respiratory virus 2 infection of the fetus results in multi-organ cell cycle suppression
Source: Vet Res. 2022 Feb 21;53:13. doi: 10.1186/s13567-022-01030-3 (PMC8860275; doi:10.1186/s13567-022-01030-3)

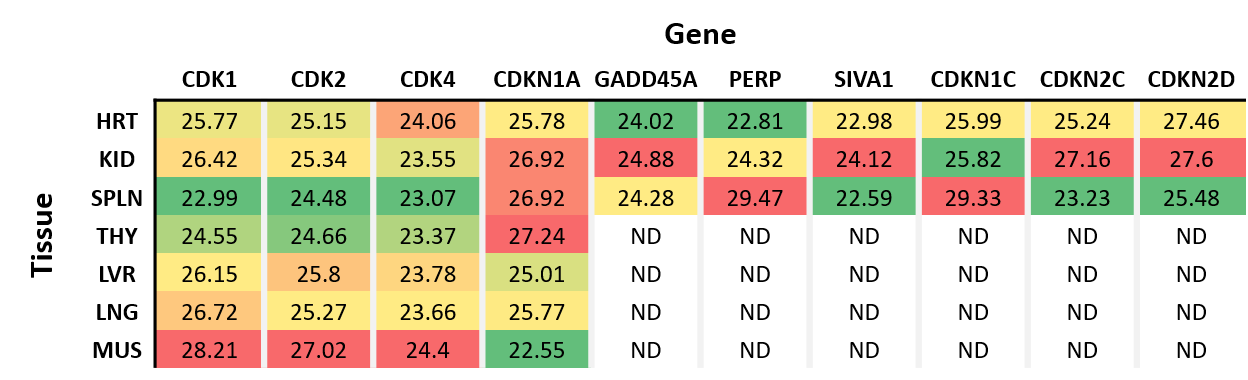

Supplement: Supplementary file 1 — Additional file 1: Raw Expression data. Average CT values across all phenotypic tissues across seven fetal organs including heart (HRT), kidney (KID), spleen (SPLN), liver (LVR), lung (LNG), thymus (THY) and loin muscle (MUS) derived from fetuses collected from Sham control and PRRS-2 challenged dams at gestation day 106 (21 days post maternal infection). Color indicates expression level within gene, with the highest expression coded green and lowest expression coded red. [file 13567_2022_1030_MOESM1_ESM.tif]

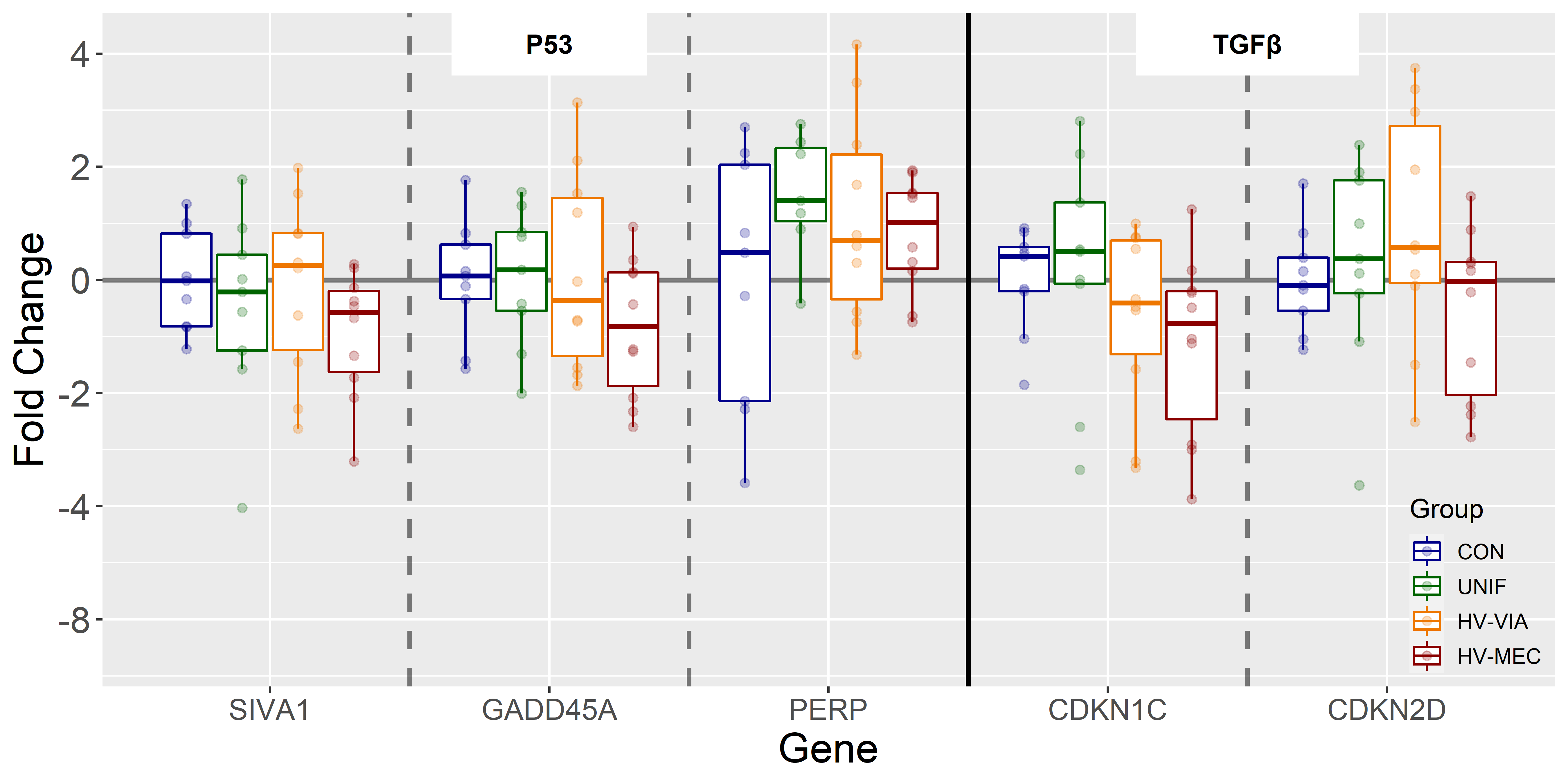

Supplement: Supplementary file 2 — Additional file 2: Splenic Gene Expression. Expression of genes known to be upregulated following activation of either P53 or TGFβ/SMAD signaling pathway in spleen derived from fetuses collected from PRRS-2 challenged dams at 21 days post-inoculation and classified based on viral load in serum and thymus as uninfected (UNIF, n = 10), high viral load viable (HV-VIA, n = 10) or high viral load meconium stained (HV-MEC, n = 10), or from control fetuses (CON n = 10) collected from gestation day matched non-inoculated control gilts. Fold changes were calculated within tissue relative to the average of the CON group. No significant differences (P < 0.05) were detected in any of the three genes in either tissue. [file 13567_2022_1030_MOESM2_ESM.tiff]

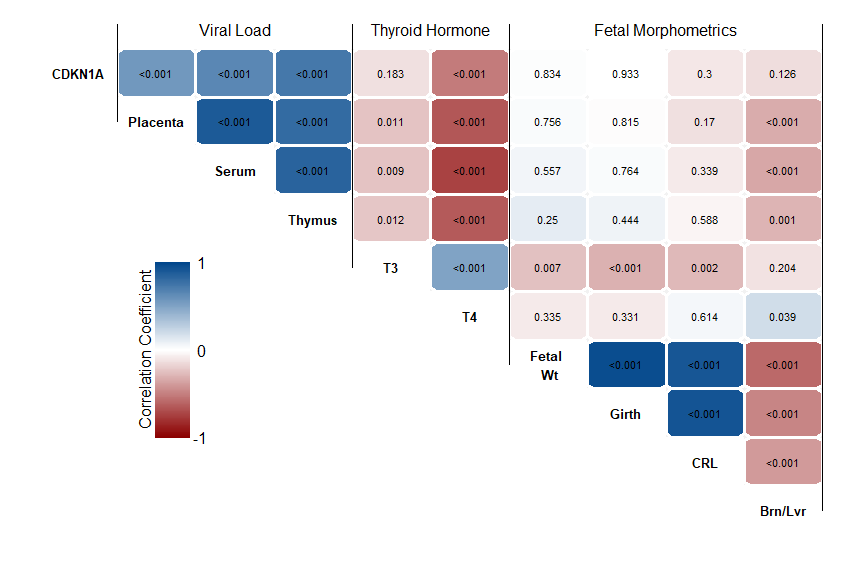

Supplement: Supplementary file 3 — Additional file 3: Phenotypic correlations with renal CDKN1A expression. Correlation heat map demonstrating the interrelationship between expression of CDKN1A in kidney tissue from n = 114 fetuses with some evidence of PRRSV infection and phenotypic parameter including viral load (placenta, serum and thymus), serum thyroid hormone concentration (T3 and T4) and fetal morphometrics. Pearson correlation coefficients were for each comparison are indicated by color with associated P value superimposed. [file 13567_2022_1030_MOESM3_ESM.tiff]
